# Parameters Identification of Fluxgate Magnetic Core Adopting the Biogeography-Based Optimization Algorithm

**DOI:** 10.3390/s16070979

**Published:** 2016-06-25

**Authors:** Wenjuan Jiang, Yunbo Shi, Wenjie Zhao, Xiangxin Wang

**Affiliations:** 1 The Higher Educational Key Laboratory for Measuring & Control Technology and Instrumentations of Heilongjiang Province, School of Measurement-Control Technology & Communications Engineering, Harbin University of Science and Technology, Harbin 150080, China; jiangwenjuan@nedu.edu.cn (W.J.); zwjsky888@163.com (W.Z.); w1073831579@126.com (X.W.); 2 School of Automation Engineering, Northeast Dianli University, Jilin 132012, China

**Keywords:** Jiles-Atherton model, biogeography-based optimization algorithm, Arnold map, differential evolution, fluxgate

## Abstract

The main part of the magnetic fluxgate sensor is the magnetic core, the hysteresis characteristic of which affects the performance of the sensor. When the fluxgate sensors are modelled for design purposes, an accurate model of hysteresis characteristic of the cores is necessary to achieve good agreement between modelled and experimental data. The Jiles-Atherton model is simple and can reflect the hysteresis properties of the magnetic material precisely, which makes it widely used in hysteresis modelling and simulation of ferromagnetic materials. However, in practice, it is difficult to determine the parameters accurately owing to the sensitivity of the parameters. In this paper, the Biogeography-Based Optimization (BBO) algorithm is applied to identify the Jiles-Atherton model parameters. To enhance the performances of the BBO algorithm such as global search capability, search accuracy and convergence rate, an improved Biogeography-Based Optimization (IBBO) algorithm is put forward by using Arnold map and mutation strategy of Differential Evolution (DE) algorithm. Simulation results show that IBBO algorithm is superior to Genetic Algorithm (GA), Particle Swarm Optimization (PSO) algorithm, Differential Evolution algorithm and BBO algorithm in identification accuracy and convergence rate. The IBBO algorithm is applied to identify Jiles-Atherton model parameters of selected permalloy. The simulation hysteresis loop is in high agreement with experimental data. Using permalloy as core of fluxgate probe, the simulation output is consistent with experimental output. The IBBO algorithm can identify the parameters of Jiles-Atherton model accurately, which provides a basis for the precise analysis and design of instruments and equipment with magnetic core.

## 1. Introduction

The fluxgate sensor is a kind of sensor which can measure a weak magnetic field by using the nonlinear relationship between the magnetic flux density B and the magnetic field strength H [1,2]. In order to analyze, design and optimize fluxgate sensor, simulations are necessary. During the simulations, the accuracy of a mathematical model for magnetic core is significant. There have been numerous approaches to model the magnetic hysteresis loop, which can be roughly divided into three categories [3]: (1) Macroscopic magnetization which ignores the underlying physics of the material behavior, such as tri-linear model, tangent function model, arctangent function model [4], and Preisach model [5] et al.; (2) Micro-magnetic theory which is derived from complex hysteresis theory but difficult to apply in real engineering materials; (3) Semi-macroscopic magnetization which combines the microstructural parameters of magnetic materials with the macroscopic magnetization curves, such as Globus model [6] and Jiles-Atherton model [7]. The Jiles-Atherton model is derived from the physical process of magnetic hysteresis and described by a first-order differential equation with five parameters. It is relatively simple in mathematical expressions and more accurate, which makes it a widely used hysteresis model in applications [8].

However, the Jiles-Atherton model is a multi-parameter model which is sensitive to the variations of parameters, so the precise determination of the model parameters has become a focus of scholars. With the development of optimization algorithms, many intelligence optimization algorithms have been proposed to identify the parameters of the Jiles-Atherton model. At present, the most frequently used methods include Genetic Algorithm (GA) [9,10], Particle Swarm Optimization (PSO) algorithm [11], Differential Evolution (DE) algorithm [12] and Simulated Annealing (SA) algorithm [13,14].

Biogeography-Based optimization (BBO) [15] is a new kind of swarm intelligence optimization algorithm, which is inspired by the study of the geographical distribution of the biological organisms. There are mainly three mechanisms in the algorithm: migration, mutation and elimination. The BBO algorithm and improved BBO algorithms have good convergence and stability, and they have been applied to solve many practical problems. Blended Biogeography-Based Optimization (B-BBO) has been applied on economic load dispatch (ELD) problem successfully [16]. Li and Yin [17] have proposed a multi-objective Biogeography-based Optimization to select the small subset of informative gene relevant to the classification. Hybridization of Biogeography-based Optimization and Ant Colony Optimization has been used to resolve mixed pixels [18]. Simulation results have shown excellent performance of these methods.

In this paper, the IBBO algorithm is used to identify the parameters of Jiles-Atherton model for magnetic core. An improved BBO (IBBO) algorithm is proposed to enhance the global searching capability, convergence rate and accuracy of the algorithm: (1) the Arnold map generating uniform chaotic sequence as original populations to improve the algorithm in ergodicity, accuracy and global searching capability; (2) introducing mutation strategy DE/best/1 of DE algorithm as mutation operator to improve the diversity of solutions and global searching capability. Applying IBBO algorithm, BBO algorithm, GA algorithm, PSO algorithm and DE algorithm to identify the Jiles-Atherton model parameters of a magnetic core at the same time, the simulation results show that the IBBO algorithm has the advantages of high precision and fast convergence rate. According to magnetic hysteresis data of the selected permalloy by experiment, the IBBO algorithm is applied to identify the parameters of the Jiles-Atherton model for the selected material. The simulation hysteresis loop is in good agreement with experimental data; the output of fluxgate probe made of above selected material as magnetic core is consistent with the simulation output. The IBBO algorithm is a new method for parameters identification of the Jiles-Atherton model.

## 2. Materials and Equipment

### 2.1. Measurement of Dynamic Hysteresis Loop

In this paper, the oscilloscope method is applied to obtain the dynamic hysteresis loop of the selected permalloy. The permalloy sample is a ring with dimensions as following: outer diameter 24 mm, inner diameter 20 mm and thickness 0.15 mm. The magnetizing field is a sinusoidal signal with frequency of 30 kHz.

**Figure 1:**
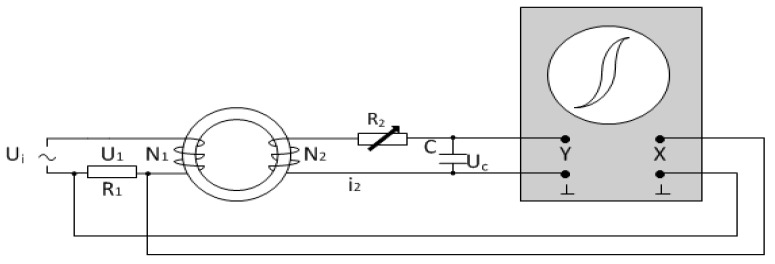
Diagram of dynamic hysteresis loop measurement.

A dynamic hysteresis loop measurement system is established according to the diagram of the measurement system shown in Figure 1 [19].

Here, R_1_ = 2.6 Ω, C = 20 μF, R_2_ = 50 kΩ, the number of excitation coils and detection coils are both 600 turns. The excitation power supply is a signal generator (AFG 3021B, TEKTRONIX, Beaverton, OR, USA) together with a power amplifier, and the oscilloscope is a digital oscilloscope (DS1052E, RIGOL, Beijing, China).

The oscilloscope method is used to measure the dynamic hysteresis loop at the excitation voltage, which is big enough to make the sample saturate. The measurement data are transported through USB port on the oscilloscope. Then the magnetic field strength H and the magnetic flux density B can be calculated according to the following equations:

(1) $$H=\frac{N_{1}\cdot U_{1}}{l\cdot R_{1}}$$

(2) $$B=\frac{R_{2}\cdot C\cdot U_{C}}{S\cdot N_{2}}$$

In Equation (2), parameter S is the cross-sectional area of the permalloy.

The B-H curve of the sample can be obtained in this way. In order to prevent the external magnetic field interfering with the sample, the measurement system is placed in a magnetic shielding cylinder (MD-10, JOZZON, Beijing, China) which is composed of six concentric cylinders. The outer layer is made of aluminum, and the other five layers are made of 1J85 permalloy.

### 2.2. Fluxgate Probe

**Figure 2:**
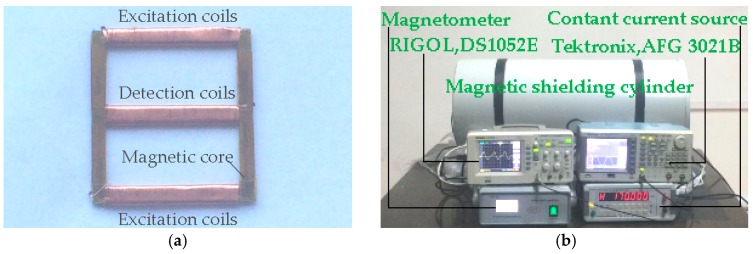
Image of fluxgate probe and measurements system. (**a**) Fluxgate probe; (**b**) Measurements of fluxgate probe.

A fluxgate probe is manufactured by the mentioned permalloy which is used as a magnetic core. The structure of fluxgate probe used in experiment is shown in Figure 2. The excitation coils are the coils both on upper and bottom arms, and the detection coils are on the middle arm. The excitation coils are 1200 turns in sum and the detection coils are 600 turns. The material of the coils is copper wire coating polyimide with the diameter of 0.15 mm.

## 3. Description of the Jiles-Atherton Model

The Jiles-Atherton model is a widely used mathematical model, which can represent the nonlinear characteristics of magnetic core accurately. The model is described by a first-order differential equation [7,20]:

(3) $$\frac{dM}{dH}=\frac{(1-c)(M_{an}-M)+ck\delta \frac{dM_{an}}{dH}}{k\delta -\alpha (1-c)(M_{an}-M)}$$

Here, *M_an_* denotes anhysteretic magnetization. *k* is pinning factor. *c* represents reversible magnetization coefficient. *α* is coupling factor between magnetic domains. *δ* is a direction parameter, which is defined as $\{\begin{array}{c} dH/dt>0,\delta =1\\ dH/dt<0,\delta =-1 \end{array}$.

The anhysteretic magnetization *M_an_* is provided by Langevin function and described in Equation (4):

(4) $$M_{an}=M_{s}\cdot \left\{coth\left[\frac{H_{e}}{a}\right]-\frac{a}{H_{e}}\right\}$$

Here, *M_s_* is saturation magnetization. Parameter *a* is form factor. *H_e_* is effective magnetic field which is given by:

*H_e_* = *H* + *αM* (5)

Combined Equations (3)–(5) can form a first order nonlinear differential equation. The numerical solution of the differential equation can be obtained when the five parameters (*M_s_, α, a, c, k*) and the initial values are determined. Thus, the calculated M-H curve can be obtained.

The model used in this paper is the original Jiles-Atherton model which describes the relationship between M and H, while the inverse Jiles-Atherton model describes the relationship between B and H. The relationship between M, B and H is following:

*B* = *μ_0_*(*M* + *H*) (6)

Francesco and Alessandro [21] have analyzed the effects of five parameters on the hysteresis loop: (1) *M_s_* mainly affects the slope dB/dH at coercive point and the maximum value of magnetization; (2) Parameter *a* affects the knee region of the loop around the remanence magnetization value; (3) *α* mainly controls the slope dB/dH in the region around the coercive point; (4) Parameters *k* and *c* control the amplitude of the loop area, the coercive field amplitude and the derivatives values on the points close to the coercive field. Table 1 [22] summarizes the influence of the parameters to the hysteresis loop. Symbols “↑” and “↓” represent increasing and decreasing respectively and symbol “--” indicates no influence or little influence.

The parameters identification of the Jiles-Atherton model is to determine the five coefficients from the experimental hysteresis loop. Utilizing appropriate optimization method, it is possible to obtain the mathematical model which has good agreement with the experimental hysteresis loop. The advantage of the Jiles-Atherton model is that the five parameters have physical meaning, which can be useful for further analysis.

## 4. The Principle of BBO Algorithm

### 4.1. Introduction of BBO Algorithm

The BBO algorithm is a new swarm intelligence optimization algorithm proposed by Simon in 2008 [15]. It is designed according to the laws of the migration, mutation and extinction of the biological species in the habitat. If a habitat is suitable for species survival, the habitat has a high fitness index (Habitat Suitability Index, HSI). HSI is related to the factors such as rainfall, humidity, temperature, and plant coverage. These factors are called Suitability Index Variables (SIVs). HSI corresponds to the fitness value of the optimized problem and the number of SIVs in each solution corresponds to the number of optimized parameters. The algorithm uses the habitat migration operator, mutation operator and elimination operator to improve the optimization capability.

#### 4.1.1. Migration Operator

BBO algorithm uses the migration operator to realize the information sharing between solutions. Each solution has its respective immigration rate and emigration rate to control the movement probabilities of solution information. The method to produce the next generation in BBO is by emigration solution features to other habitats and receiving solution features by immigration from other habitats. The algorithm assumes a high species count in habitats having high HSI, which encourages species to emigrate from the habitat, sharing their SIVs with other habitats. Hence, habitats with high HSI have high emigration rate and low immigration rate. Like other swarm based algorithms, the BBO algorithm replaces SIVs of emigration solution with SIVs of immigration solution in iterations to increase the diversity of swarms and the precision of solutions.

#### 4.1.2. Mutation Operator

Affected by emergencies, some environmental indicators of habitat have changed suddenly, which can be simulated by the mutation operator.

Simon has reported that the mutation rate in the habitat is inversely proportional to the species count probability, which can be described as follows:

(7) $$m_{s}=m_{\max}\left[\frac{1-p_{s}}{p_{\max}}\right]$$

Here, *m_s_* is mutation rate of a habitat with s number of species, *m_max_* is maximum mutation rate defined by user, *p_s_* is probability of a habitat with s number of species and *p_max_* is the maximum of *p_s_*.

Mutation can increase the probability of the mutation of SIVs of low HSI, and improve the HSI, but the SIVs of high HSI may be destroyed at the same time. So the strategy of elite reservation is introduced in the iteration process.

#### 4.1.3. Elimination Operator

In order to avoid the homogeneous solution and improve the diversity of population, Simon has designed elimination operator to clear the same solutions and replace the same solutions by randomly generated solutions.

### 4.2. Improved BBO Algorithm

In the process of iterations, BBO algorithm has poor global search capability, low accuracy and convergence rate [23,24]. The following improvements are made to the BBO algorithm to increase global search capability and improve the accuracy of solutions and convergence rate.

#### 4.2.1. Generating Initial Populations by Arnold Map

Chaos is a kind of nonlinear phenomenon in nature. It has the characteristics of randomicity, ergodicity and regularity, which can improve the searching capability of the algorithm [25].

In practice, the Logistic chaotic sequence is often introduced into the algorithms to improve the optimization capability. However, the distribution of the Logistic map is uneven, and has strong boundary search capability and low middle search probability. So it is poor in ergodicity [26].

The sequence generated by the Arnold map is uniform and sensitive to initial value. So in this paper, the Arnold map is used to generate initial populations of BBO to improve the ergodicity and global searching capability of the algorithm. In this paper, the five parameters need to be determined, so the five-dimensional Arnold map is used to generate uniform chaotic sequence. The five-dimensional Arnold map equation is as follows [27]:

(8) $$\left[\begin{array}{c} x_{n+1}\\ y_{n+1}\\ z_{n+1}\\ k_{n+1}\\ l_{n+1} \end{array}\right]=\left[\begin{array}{ccccc} 1 & 1 & 1 & 1 & 1\\ 1 & 2 & 2 & 2 & 2\\ 1 & 2 & 3 & 3 & 3\\ 1 & 2 & 3 & 4 & 4\\ 1 & 2 & 3 & 4 & 5 \end{array}\right]\left[\begin{array}{c} x_{n}\\ y_{n}\\ z_{n}\\ k_{n}\\ l_{n} \end{array}\right](mod\;N)$$

Here, *modN* represents the fractional part, *n* is a positive integer, and the values of *x_n_, y_n_...l_n_* are all in [0, 1].

A uniform chaotic sequence generated by Arnold map is selected as initial populations of BBO algorithm, instead of Rand random function. The distribution of the initial populations is more uniform, which increases the accuracy of the solution and the searching capability of the algorithm.

#### 4.2.2. Improving Migration Equation by DE Mutation Strategy

In the migration process, BBO algorithm replaces SIVs of emigration solution with SIVs of immigration solution. The migration equation of the conventional BBO algorithm is:

*H_i_(SIV)* = *H_j_(SIV)* (9)

The migration Equation (9) is simple, which makes the new solution has only a small amount of information and affects the performance of the BBO algorithm. In order to solve this problem, the DE/best/1 mutation strategy of DE algorithm [28] is introduced to the migration equation:

(10) $$H_{i}(SIV)=H_{best}(SIV)+F[H_{r1}(SIV)-H_{r2}(SIV)]$$

Here, *H_best_* is the best optimal solution, *H_r1_* and *H_r2_* are two different solutions randomly selected, and *F* is a scaling factor with the range of [0, 1].

Equation (10) shows that the solution preserves the information of the best optimal solution and absorbs the differential vector of the other solutions after migration. Scaling factor *F* balances the exploitation and exploration ability of the algorithm, which can avoid homogeneity of solution and increase the evolution rate of the excellent solutions.

### 4.3. Steps of Identification the Jiles-Atherton Model Parameters with IBBO

The steps of using IBBO to identify the parameters of Jiles-Atherton model are as follows:

Step 1: Set the initial values of IBBO algorithm and the range of the parameters of the Jiles-Atherton model.

Step 2: Apply the Arnold map to generate the initial solutions of the Jiles-Atherton model.

Step 3: Calculate the HSI values of the solutions, sort the HSI values in descending order (for minimum problems), and keep the optimal solutions. The fitness function is mentioned in Section 5.

Step 4: According to the iteration termination conditions, determine whether the iteration is terminated. If the termination conditions are satisfied, output the optimal solution; if not, continue the following steps.

Step 5: Use the cosine model to calculate the immigration probability and the emigration probability of each habitat, and apply the mutation strategy of DE/best/1 to improve the migration equation.

Step 6: According to mutation probability, operate mutation of the population, which improves the diversity of the solution.

Step 7: Compare one solution with the others, find the same solutions, replace one of the same solutions with a randomly generated solution, and return to Step 3 to next generation iteration of the solutions.

**Figure 3:**
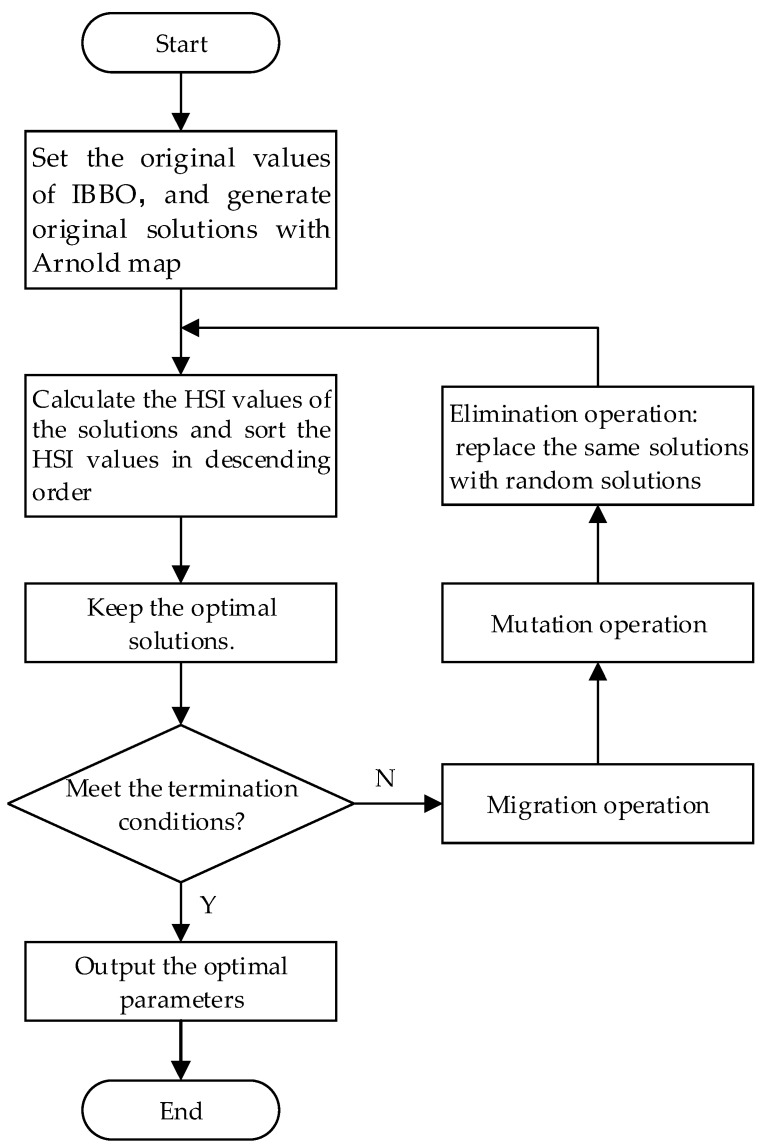
Flow chart of improved Biogeography-Based Optimization (IBBO).

The flow chart of IBBO algorithm to identify the parameters of Jiles-Atherton model is shown in Figure 3.

## 5. Simulation Results and Analysis

### 5.1. Simulation Results for Theoretical Data

In this paper, a set of data is selected as the parameters of the Jiles-Atherton model. The hysteresis data can be obtained by solving the Equations (3)–(5) with 4th/5th Runge-Kutta method. In this case, the experimental data is replaced by numerical solutions. According to the numerical solutions, the parameters of the Jiles-Atherton model are determined by IBBO method. Also, the GA method, PSO method, DE method and BBO method are used to identify the parameters.

The simulation parameters of five algorithms are set as following:

(1) IBBO: population size is 20, iteration number is 50, initial mutation probability is 0.005 and scaling factor is 0.5.
(2) BBO: population size is 20, iteration number is 50, initial mutation probability is 0.005.
(3) GA: population size is 20, the maximum genetic algebra is 50, the binary number of variables is 20, mutation probability is 0.05, crossover probability is 0.9.
(4) PSO: particle swarm size is 20, the maximum number of iterations is 50, the inertia factor is 0.6, and the acceleration constants c_1_ = c_2_ = 2.
(5) DE: population size is 20, the maximum number of iterations is 50, mutation probability is 0.6, and crossover probability is 0.8.

The above algorithms are all optimization algorithms, so an optimized function (fitness function) must be defined. The optimized parameters are the five parameters (*M_s_,* *α, a, c, k*), so the fitness function must be described by the five parameters. Once the five parameters are determined, a set of solutions can be obtained by solving the Equations (3)–(5).The smaller the difference between the calculated B and experimental B at the same H is, the more accurate the parameters are. In this paper, the fitness function is defined as follows:

(11) $$fitness=\sqrt{\frac{1}{N}\sum_{i=1}^{N}{\left(B_{expi}-B_{i}\right)}^{2}}$$

Here, *B_expi_* is experimental data, and *B_i_* is calculated (simulation) data.

The five algorithms are run for 30 times respectively. The simulation results are listed in Table 2. The results in Table 2 are averaged. From Table 2, it is found that: the fitness value of IBBO method is 0.0225 T which is the minimum fitness value of the five methods, and the simulation time of IBBO method is 197.25 s which is 25% faster than those of GA and PSO methods and more than 2 times faster than that of DE method. It is stated that the IBBO method runs faster and is more accurate compared with BBO, GA, PSO, and DE methods, due to the introduction of the Arnold map and DE mutation strategy.

**Figure 4:**
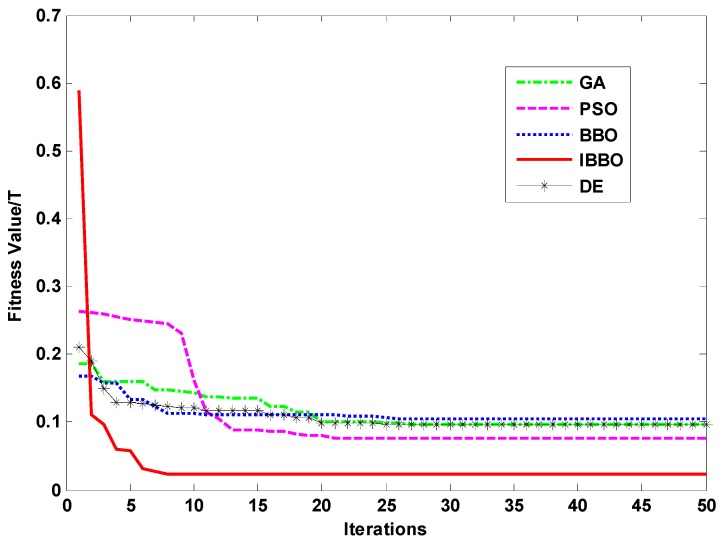
Optimization process of fitness function.

Figure 4 shows the fitness values with iterations of five methods. IBBO method needs 7 iterations to converge to the best fitness value, which is faster than those of GA, PSO and BBO methods. The IBBO method has the smallest fitness value of the five methods. It can be seen that IBBO method has high accuracy and convergence rate in the identification of the parameters for the Jiles-Atherton model.

**Figure 5:**
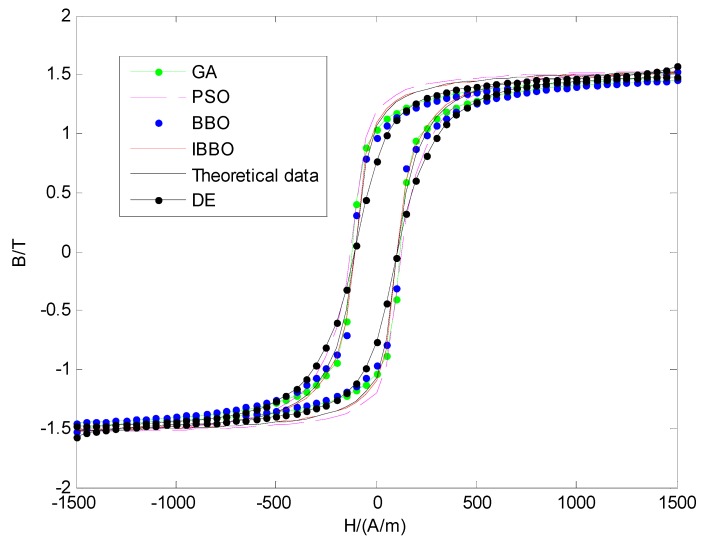
Hysteresis Loops for five different methods.

The five hysteresis loops obtained with the parameters in Table 2 are shown in Figure 5. It can be found that the hysteresis loop achieved by IBBO method is in high agreement with the theoretical data.

**Figure 6:**
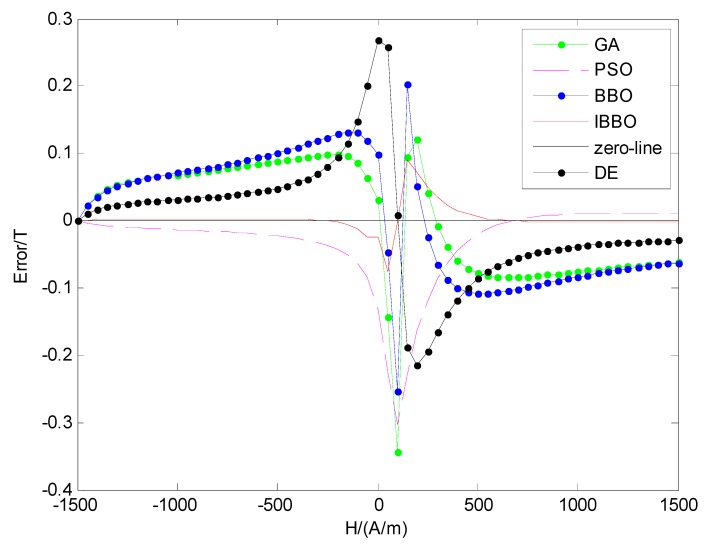
Errors of five methods on ascending part.

The errors between the simulation data and the theoretical data are shown in Figure 6. From Figure 6, it can be found that the maximum absolute error of the red line (obtained by IBBO) is 0.091 T at 150 A/m on the ascending part; the maximum absolute errors of the blue line (obtained by BBO) and the green line (obtained by GA) are 0.2535 T and 0.3443 T at 100 A/m respectively; the maximum absolute errors of PSO method and DE method are 0.3037 T and 0.2675 T; the errors are mainly concentrated on the middle part of H around coercive force point H_c_. It is stated that the identification accuracy of IBBO method is the best. It can be used to determine the parameters of Jiles-Atherton model accurately.

### 5.2. Simulation Results for Experimental Data

**Figure 7:**
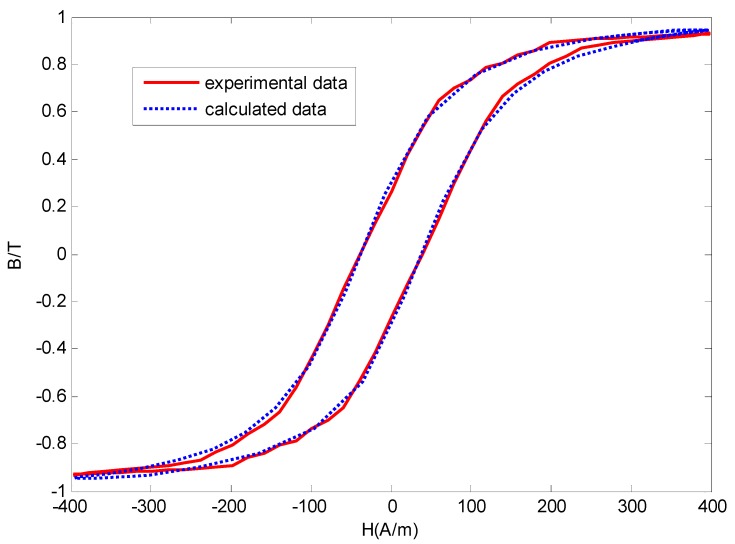
Experimental B-H hysteresis Loop and calculated B-H hysteresis Loop obtained by IBBO.

According to the principle of dynamic hysteresis loop measurement, the experimental hysteresis loop is obtained, as shown in Figure 7. Also the five methods are applied to identify the parameters of Jiles-Atherton model. Based on the influence of the parameters to hysteresis loop, the range of the parameters are determined, as seen in Table 3 together with the identification results. From Table 3, the smallest fitness value is 0.0544 T which is obtained by IBBO method; the biggest fitness value is 0.1707 T obtained by GA method; the fitness values of PSO, DE and BBO are in the middle. The simulation time of IBBO method is 199.03 s which is 25% faster than those of GA and PSO methods and 2.4 times faster than that of DE method. So the IBBO method has the advantage of fast convergence rate and good accuracy.

The simulation hysteresis loop obtained by IBBO is also depicted in Figure 7, from which it can be found that the simulation hysteresis loop is in high agreement with the experimental one.

**Figure 8:**
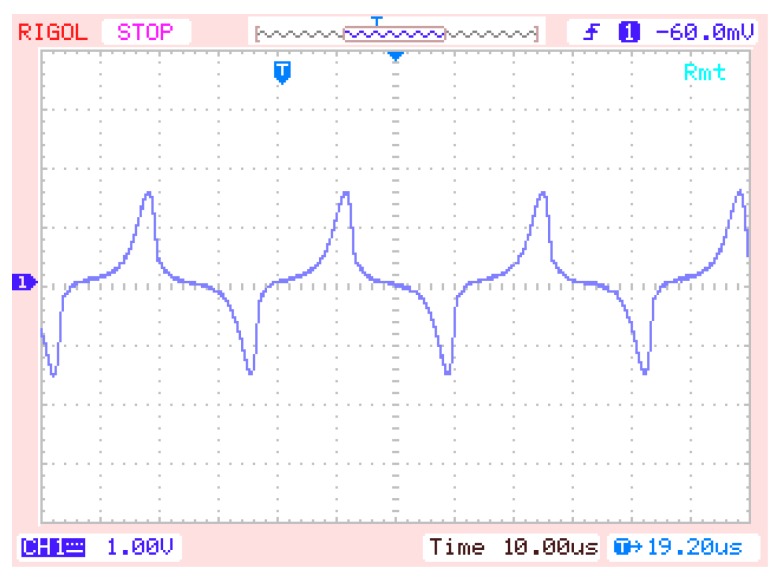
Output of fluxgate probe.

The experiment is carried out on fluxgate probe using the mentioned permalloy as the magnetic core. The structure of the magnetic fluxgate probe used in the experiment is depicted in Figure 2, which is completely symmetrical in structure. When the external magnetic field is 30,000 nT, the experimental output of the magnetic fluxgate probe is shown in Figure 8. The output waveform is a periodic signal with period T = 33.6 μs, the maximum value V_max_ = 1.52 V and the minimum value V_min_ = −1.6 V.

**Figure 9:**
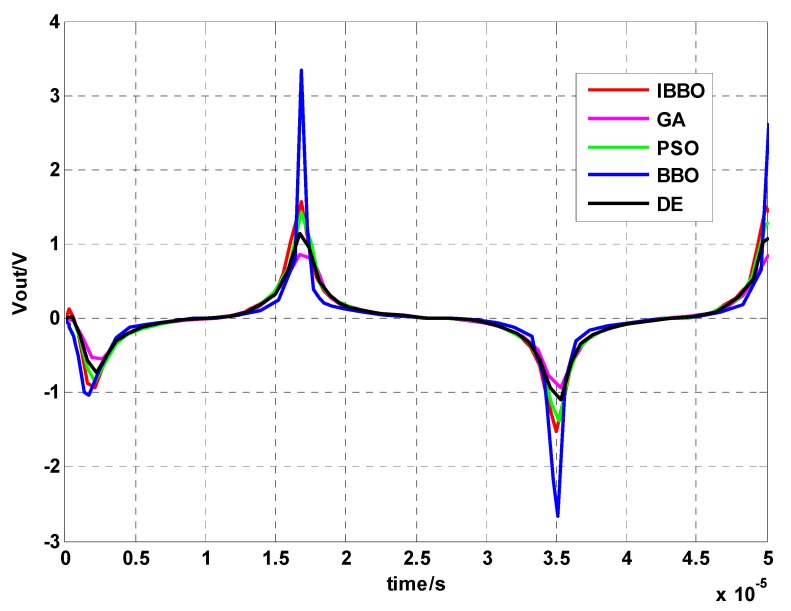
Simulation outputs of fluxgate probe.

The fluxgate probe is a single-rod sensor in theory. A simulation model based on the Jiles-Atherton model is built in a MATLAB/Simulink environment. Figure 9 shows the simulation outputs of different parameters optimized by five methods. The output waveforms are periodic signals with period T = 33.38 μs. The maximum values V_max_ for IBBO method, BBO method, DE method, PSO method and GA method are 1.57 V, 3.33 V, 1.13 V, 1.41 V and 0.85 V respectively, and the minimum values V_min_ are −1.53 V, −2.65 V, −1.09 V, −1.39 V, and −0.94 V respectively. The simulation output of parameters optimized by IBBO method is consistent with the experimental output of the fluxgate probe.

## 6. Conclusions

In this paper, the BBO algorithm is applied to identify the five parameters of the Jiles-Atherton model. Through the improvement of the BBO algorithm, the convergence rate and the optimization accuracy are improved. Using the IBBO algorithm to identify the fixed parameters of the Jiles-Atherton model, simulation results have shown that IBBO algorithm has been improved significantly in terms of convergence rate and accuracy compared with GA, PSO, DE and BBO algorithms. For the permalloy Jiles-Atherton model parameters identification, the hysteresis loop of simulation and experiment are in high agreement. The simulation output of parameters optimized by IBBO method is consistent with the experimental output. IBBO method is a new approach for parameter identification of the Jiles-Atherton model, which provides a reference for further accurate analysis and design of the instruments and equipment with a magnetic core.

## Figures and Tables

**Table 1:** Influence of the parameters to the hysteresis loop.

Parameters	Remanence	Coercive Force	dB/dH at Coercive Point	Loop Area	Maximum Magnetization
*M_s_*_↑_	_↑_	--	_↑_	--	_↑_
*α*_↑_	_↑_	--	_↑_	--	--
*a*_↑_	_↓_	--	_↓_	--	--
*c*_↑_	_↓_	_↓_	--	_↓_	--
*k*_↑_	_↑_	_↑_	--	_↑_	--

**Table 2:** Identification results of fixed parameters.

Parameters	GA	PSO	DE	BBO	IBBO	Theoretical Data
*M_s_* × 10^6^ (A/m)	1.2346	1.2416	1.2348	1.2337	1.2423	1.24
*α* × 10^−5^	6.6773	8.3791	4.4841	6.9382	7.2841	9.18
*a* (A/m)	48.69	48.51	63.569	45.75	49.68	54
*c*	0.686	0.464	0.751	0.611	0.318	0.338
*k* (A/m)	114.91	115.24	120.67	122.85	117.12	119.36
Fitness value	0.096	0.0765	0.0963	0.105	0.0225	--
simulation time (s)	241.91	256.53	654.21	201.36	197.25	--

**Table 3:** Identification results of permolloy.

Parameters	Range	GA	PSO	DE	BBO	IBBO
*M_s_* × 10^6^ (A/m)	[0.95, 1.05]	0.9552	1.0221	0.9758	0.9738	1.0359
*α* × 10^−5^	[10^−3^, 10^2^]	40.957	32.546	35.648	39.482	30.551
*a* (A/m)	[1, 200]	196.73	140.51	170.56	145.75	148.79
*c*	[0, 1]	0.5423	0.4641	0.4561	0.6112	0.3876
*k* (A/m)	[30, 80]	41.321	45.244	43.234	38.852	39.512
Fitness value (T)	--	0.1707	0.0613	0.0682	0.0756	0.0544
simulation time (s)	--	251.43	279.92	678.94	210.67	199.03
